# Outcomes of basilar artery occlusion in patients aged 75 years or older in the Basilar Artery International Cooperation Study

**DOI:** 10.1007/s00415-012-6498-2

**Published:** 2012-04-18

**Authors:** Mervyn D. I. Vergouwen, Annette Compter, David Tanne, Stefan T. Engelter, Heinrich Audebert, Vincent Thijs, Gabriel de Freitas, Ale Algra, L. Jaap Kappelle, Wouter J. Schonewille

**Affiliations:** 1 Department of Neurology and Neurosurgery, UMC Utrecht Stroke Center, Rudolf Magnus Institute of Neurosciences, University Medical Center Utrecht, Heidelberglaan 100, 3584 CX Utrecht, The Netherlands; 2 Department of Neurology, Stroke Center, Chaim Sheba Medical Centre, Tel Aviv University, Tel-Hashomer, Israel; 3 Department of Neurology, University Hospital Basel, Basel, Switzerland; 4 Center for Stroke Research, Charité University Medicine Berlin, Campus Benjamin, Franklin, Germany; 5 Department of Neurology, University Hospitals Leuven and Vesalius Research Centre, VIB, Leuven, Belgium; 6 Department of Neurology, University of Rio de Janeiro, Rio de Janeiro, Brazil; 7 Julius Center for Health Sciences and Primary Care, University Medical Center, Utrecht, The Netherlands; 8 Department of Neurology, St. Antonius Hospital, Nieuwegein, The Netherlands

**Keywords:** Basilar artery occlusion, Age, Recanalization, Outcome, Mortality

## Abstract

Patients with an acute basilar artery occlusion (BAO) have a high risk of long-lasting disability and death. Only limited data are available on functional outcome in elderly patients with BAO. Using data from the Basilar Artery International Cooperation Study, we aimed to determine outcomes in patients ≥75 years. Primary outcome measure was poor functional outcome (modified Rankin scale score 4–6). Secondary outcomes were death, insufficient vessel recanalization (defined as thrombolysis in myocardial infarction score 0–1) and symptomatic intracranial hemorrhage (SICH). Patients were divided into four age-groups, based on quartiles: 18–54, 55–64, 65–74, and ≥75 years. Outcomes were compared between patients ≥75 years and patients aged 18–54 years. Risk ratios with corresponding 95 % confidence intervals (CI) were calculated and Poisson regression analyses were performed to calculate adjusted risk ratios (aRR). We included 619 patients [18–54 years *n* = 153 (25 %), 55–64 years *n* = 133 (21 %), 65–74 years *n* = 171 (28 %), and ≥75 years *n* = 162 (26 %)]. Compared with patients aged 18–54 years, patients ≥75 years were at increased risk of poor functional outcome [aRR 1.33 (1.14–1.55)] and death [aRR 2.47 (1.75–3.51)]. Nevertheless, 35/162 (22 %, 95 % CI 15–28 %) of patients ≥75 years had good functional outcome. No significant differences between age groups were observed for recanalization rate and incidence of SICH. Although patients ≥75 years with BAO have an increased risk of poor outcome compared with younger patients, a substantial group of patients ≥75 years survives with a good functional outcome.

**Electronic supplementary material:**

The online version of this article (doi:10.1007/s00415-012-6498-2) contains supplementary material, which is available to authorized users.

## Introduction

Patients with an acute basilar artery occlusion (BAO) have a high risk of long-lasting disability and death [1, 2]. Although higher age, analyzed as a continuous variable, has been associated with poor functional outcome after BAO, only limited data are available on functional outcome in elderly patients [3–8]. One small case series suggested that all patients ≥75 years have poor functional outcome [6]. In another study, the eldest surviving patient in whom recanalization was successful was 63-year-old [3]. We analysed data from the Basilar Artery International Cooperation Study (BASICS) to determine outcomes in patients with BAO ≥75 years.

## Methods

BASICS is a prospective, observational, registry of 619 consecutive patients ≥18 years with an acute symptomatic BAO [2, 9]. The protocol was approved by the ethics committee of the University Medical Center Utrecht, the Netherlands. Embolic BAO was defined as complete recanalization on follow-up and no indication of dissection, or maximum deficit from onset and cardiac or vertebral source of embolism, or maximum deficit from onset with complete absence of other atherosclerotic cerebrovascular lesions. Atherosclerotic BAO was defined as known symptomatic basilar artery stenosis (>50 %) prior to occlusion, or residual stenosis after recanalization and no evidence of cardiac or vertebral artery source of embolism, or prior TIAs or stroke in the basilar artery territory and no evidence of cardiac or vertebral artery source of embolism. Dissections were not predefined, but scored according to the investigators.

Primary outcome was poor functional outcome after 1 month [predefined as modified Rankin scale (mRS) score 4–6]. Secondary outcomes were death, insufficient vessel recanalization [defined as thrombolysis in myocardial infarction (TIMI) score 0–1] and symptomatic intracranial hemorrhage (SICH). We also investigated if our conclusions changed if poor functional outcome was defined as an mRS of 3–6. SICH was not predefined by the registry, and the reporting of SICH was done on the basis of each investigator’s judgment. For the purpose of this study, patients were divided into four age-groups, based on quartiles: 18–54, 55–64, 65–74, and ≥75 years. Outcomes were compared between patients ≥75 years and patients aged 18–54 years. Risk ratios (RR) and corresponding 95 % confidence intervals (CI) were calculated. Variables that affected the crude risk ratio most were used simultaneously in Poisson regression analyses to calculate adjusted risk ratios (aRR) [2]. Missing baseline data (<5 % for each variable) were imputed with regression imputation [10]. Finally, we explored the incidence of poor functional outcome in patients 75–79, 80–84, 85–89, and 90 years or older.

## Results

Baseline characteristics are listed in Table 1. In total, 162 patients (26 % of total cohort) were ≥75 years. In this group of patients, the most common cause of stroke was embolism and 64 % had an NIHSS score >20. Treatment of any kind was initiated in 148 patients (91 %).

**Table 1:** Baseline characteristics according to age group

Variable	All patients *n* = 619	18–54 years *n* = 153	55–64 years *n* = 133	65–74 years *n* = 171	≥75 years *n* = 162
Male sex	390 (63 %)	94 (61 %)	105 (79 %)	116 (68 %)	75 (46 %)
Hypertension	383 (62 %)	41 (27 %)	87 (65 %)	133 (78 %)	122 (75 %)
Diabetes mellitus	135 (22 %)	13 (8 %)	31 (23 %)	49 (29 %)	42 (26 %)
Hyperlipidemia	167 (27 %)	30 (20 %)	45 (34 %)	55 (32 %)	37 (23 %)
Atrial fibrillation	133 (21 %)	3 (2 %)	20 (15 %)	42 (25 %)	68 (42 %)
Coronary artery disease	109 (18 %)	12 (8 %)	21 (16 %)	35 (20 %)	41 (25 %)
Location of basilar artery occlusion					
Distal third	202 (33 %)	42 (27 %)	30 (23 %)	55 (32 %)	75 (46 %)
Middle third	143 (23 %)	37 (24 %)	29 (22 %)	46 (27 %)	31 (19 %)
Proximal third	274 (44 %)	74 (48 %)	74 (56 %)	70 (41 %)	56 (35 %)
Median NIHSS score (IQR)	22 (12–30)	20 (9–30)	20 (12–30)	22 (13–28)	25 (16–30)
NIHSS score >20	336 (54 %)	73 (48 %)	66 (50 %)	93 (54 %)	104 (64 %)
Type of treatment					
Antithrombotics	183 (30 %)	47 (31 %)	33 (25 %)	52 (30 %)	51 (31 %)
IV tPA or IV tPA/IAT	121 (20 %)	25 (16 %)	37 (28 %)	24 (14 %)	35 (22 %)
IAT only	288 (47 %)	78 (51 %)	59 (44 %)	89 (52 %)	62 (38 %)
No treatment	27 (4 %)	3 (2 %)	4 (3 %)	6 (4 %)	14 (9 %)
Time to treatment					
0–3 h	179 (29 %)	41 (27 %)	39 (29 %)	49 (29 %)	50 (31 %)
4–6 h	190 (31 %)	45 (29 %)	37 (28 %)	58 (34 %)	50 (31 %)
7–9 h	84 (14 %)	22 (14 %)	19 (14 %)	26 (15 %)	17 (10 %)
10–12 h	57 (9 %)	14 (9 %)	14 (11 %)	16 (9 %)	13 (8 %)
13–24 h	55 (9 %)	17 (11 %)	15 (11 %)	9 (5 %)	14 (9 %)
>24 h	27 (4 %)	11 (7 %)	5 (4 %)	7 (4 %)	4 (2 %)
Cause of stroke					
Embolism	224 (36 %)	42 (27 %)	38 (29 %)	59 (35 %)	85 (52 %)
Atherosclerosis	215 (35 %)	39 (25 %)	57 (43 %)	72 (42 %)	47 (29 %)
Dissection	32 (5 %)	24 (16 %)	5 (4 %)	3 (2 %)	0 (0 %)
Other	6 (1 %)	2 (1 %)	1 (1 %)	2 (1 %)	1 (1 %)
Unknown	142 (23 %)	44 (29 %)	30 (23 %)	31 (18 %)	27 (17 %)

*NIHSS* national institutes of health stroke scale, *IQR* interquartile range, *IV* intravenous, *tPA* tissue plasminogen activator, *IAT* any intra-arterial treatment (either intra-arterial tPA, mechanical clot disruption, or both)

**Fig. 1:**
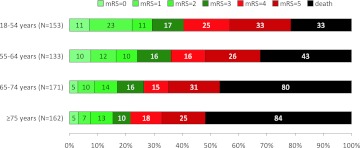
Modified Rankin scale scores according to age group. *mRS* modified Rankin scale score

Modified Rankin Scale scores for all age groups are presented in Fig. 1. Patients ≥75 years had a higher risk of poor functional outcome [aRR 1.33 (1.14–1.55), Table 2] and death [aRR 2.47 (1.75–3.51), Table 3] than patients aged 18–54 years. Nevertheless, 35 patients (22 %, 95 % CI 15–28 %) of those ≥75 years had a good functional outcome. No significant differences between age groups were observed for insufficient recanalization [patients ≥75 vs. 18–54 years aRR 1.69 (0.95–3.03)] and SICH [patients ≥75 vs. 18–54 years RR 1.77 (0.77–4.06)]. Since SICH occurred in only 42 (7 %) patients, no further analyses were performed for this outcome measure.

**Table 2:** Poor functional outcome (mRS 4–6) according to age-group

	18–54 years^a^	55–64 years	65–74 years	≥75 years
Total	153 (25 %)	133 (21 %)	171 (28 %)	162 (26 %)
Poor outcome	91/153 (60 %)	85/133 (64 %)	126/171 (74 %)	127/162 (78 %)
Unadjusted	1	1.08 (0.90–1.29)	1.24 (1.06–1.45)	1.32 (1.13–1.54)
Adjusted				
Male sex	1	1.06 (0.88–1.28)	1.23 (1.05–1.45)	1.33 (1.14–1.55)
Location of occlusion	1	1.06 (0.89–1.27)	1.26 (1.08–1.47)	1.37 (1.18–1.60)
NIHSS	1	1.06 (0.90–1.25)	1.22 (1.06–1.41)	1.23 (1.06–1.41)
Time to treatment (h)	1	1.08 (0.90–1.30)	1.26 (1.07–1.49)	1.33 (1.13–1.56)
Type of treatment	1	1.07 (0.89–1.29)	1.24 (1.05–1.46)	1.32 (1.12–1.55)
3 factors^b^	1	1.05 (0.89–1.24)	1.24 (1.07–1.43)	1.28 (1.11–1.48)
5 factors^c^	1	1.06 (0.90–1.25)	1.28 (1.10–1.49)	1.33 (1.14–1.55)

^a^Reference group. Data are number (%) or risk ratio (95 % CI)

^b^Adjustment for national institutes of health stroke scale (NIHSS) score, sex, and location of occlusion

^c^Adjustment for NIHSS score, sex, location of occlusion, time to treatment, and type of treatment

**Table 3:** Mortality according to age group

	18–54 years^a^	55–64 years	65–74 years	≥75 years
Total	153 (25 %)	133 (21 %)	171 (28 %)	162 (26 %)
Mortality	33/153 (22 %)	43/133 (32 %)	80/171 (47 %)	84/162 (52 %)
Unadjusted	1	1.50 (1.02–2.21)	2.17 (1.54–3.05)	2.40 (1.72–3.37)
Adjusted				
Male sex	1	1.48 (1.002–2.19)	2.16 (1.53–3.04)	2.43 (1.73–3.40)
Location of occlusion	1	1.47 (0.998–2.16)	2.22 (1.58–3.11)	2.55 (1.83–3.56)
NIHSS	1	1.46 (1.008–2.12)	2.13 (1.55–2.94)	2.16 (1.57–2.98)
Time to treatment (h)	1	1.56 (1.04–2.35)	2.28 (1.59–3.27)	2.41 (1.68–3.46)
Type of treatment	1	1.55 (1.03–2.34)	2.24 (1.56–3.21)	2.42 (1.68–3.47)
3 factors^b^	1	1.47 (1.02–2.12)	2.22 (1.61–3.05)	2.31 (1.68–3.17)
5 factors^c^	1	1.56 (1.05–2.31)	2.41 (1.71–3.39)	2.47 (1.75–3.51)

^a^Reference group. Data are number (%) or risk ratio (95 % CI)

^b^Adjustment for national institutes of health stroke scale (NIHSS) score, sex and location of occlusion

^c^Adjustment for NIHSS score, sex, location of occlusion, time to treatment, and type of treatment

If poor functional outcome was defined as an mRS of 3–6, the proportion of patients with poor functional outcome in each age group was 108/153 (71 %) in patients 18–54 years of age, 101/133 (76 %) in patients 55–64 years, 142/171 (83 %) in patients 65–74 years, and 137/162 (85 %) in patients ≥75 years. Also when this definition was used, patients ≥75 years had a higher risk of poor functional outcome [aRR 1.21 (1.07–1.36)] than patients aged 18–54 years.

Baseline characteristics of patients ≥75 years and their relationship with functional outcome are shown in Table 4. The following variables were associated with an increased risk of poor functional outcome after 1 month: male sex (RR 1.18, 95 % CI 1.00–1.38), location of occlusion (middle third vs. distal third: RR 1.21, 95 % CI 1.02–1.44), NIHSS score >20 on presentation (RR 1.36, 95 % CI 1.10–1.67), type of treatment (no treatment vs. intra-arterial thrombolytic therapy (IAT) only: RR 1.32, 95 % CI 1.15–1.52), SICH (RR 1.21, 95 % CI 1.03–1.43), and insufficient recanalization (RR 1.38, 95 % CI 1.07–1.77).

**Table 4:** Characteristics of patients ≥75 years and risk of poor functional outcome

Variable	Risk of poor functional outcome (mRS 4–6)		RR (95 % CI)
	Characteristic present	Characteristic absent	
Male sex	64/75 (85 %)	63/87 (72 %)	1.18 (1.00–1.38)
Hypertension	98/122 (80 %)	29/40 (73 %)	1.11 (0.90–1.37)
Diabetes mellitus	29/42 (69 %)	98/120 (82 %)	0.85 (0.68–1.05)
Hyperlipidemia	27/37 (73 %)	100/125 (80 %)	0.91 (0.74–1.13)
Atrial fibrillation	57/68 (84 %)	70/94 (74 %)	1.13 (0.96-1.32)
Coronary artery disease	35/41 (85 %)	92/121 (76 %)	1.12 (0.96–1.32)
Location of basilar artery occlusion^a^			
Proximal third	43/56 (77 %)	56/75 (75 %)	1.03 (0.85–1.25)
Middle third	28/31 (90 %)	56/75 (75 %)	1.21 (1.02–1.44)
NIHSS score >20	90/104 (87 %)	37/58 (64 %)	1.36 (1.10–1.67)
Type of treatment^b^			
Antithrombotics	38/51 (75 %)	47/62 (76 %)	0.98 (0.79–1.22)
IV tPA or IV tPA/IAT	28/35 (80 %)	47/62 (76 %)	1.06 (0.85–1.31)
No treatment	14/14 (100 %)	47/62 (76 %)	1.32 (1.15–1.52)
Time to treatment^c^			
4–6 h	35/50 (70 %)	38/50 (76 %)	0.92 (0.73–1.17)
7–9 h	13/17 (76 %)	38/50 (76 %)	1.01 (0.74–1.37)
≥10 h	27/31 (87 %)	38/50 (76 %)	1.15 (0.93–1.41)
Cause of stroke			
Embolism^d^	65/85 (76 %)	38/47 (81 %)	0.95 (0.79–1.14)
Symptomatic intracranial hemorrhage	14/15 (93 %)	113/147 (77 %)	1.21 (1.03–1.43)
Insufficient recanalization	18/20 (90 %)	32/49 (65 %)	1.38 (1.07–1.77)

^a^Distal occlusion was taken as reference and is recorded under “characteristic absent”

^b^IAT only was taken as reference and is recorded under “characteristic absent”

^c^0–3 h was taken as reference and is recorded under “characteristic absent”

^d^Atherosclerosis was taken as reference and is recorded under “characteristic absent”

The proportion of patients with poor functional outcome in age subgroups ≥75 years was as follows: 64/82 (78 %) in patients 75–79 years of age, 40/49 (82 %) in patients 80–84 years, 16/23 (70 %) in patients 85–89 years, and 7/8 (88 %) in patients 90 years or older. The oldest patient with good functional outcome was 91 years of age, despite an NIHSS score of 21 on admission. This patient was treated with intravenous recombinant tissue plaminogen activator (rtPA) only, and had an mRS score of 2 at 1 month follow-up.

## Discussion

The BASICS study shows that patients ≥75 years with BAO have an increased risk of poor functional outcome and death compared with younger patients, despite comparable recanalization rates. In contrast with a small previous study [6], our data show that a substantial group of patients ≥75 years survives with good functional outcome.

Previously, it has been suggested that the increased risk of poor functional outcome in elderly patients resulted from a higher prevalence of atherosclerotic occlusions and consequently lower recanalization rates [3]. However, in our study population patients ≥75 years were more likely to have an embolic rather than an atherosclerotic cause of BAO, mainly due to a higher prevalence of atrial fibrillation. Patients ≥75 years with an embolic cause of BAO had a similar risk of poor functional outcome compared with patients in this age group with an atherosclerotic cause of BAO. Sufficient recanalization was achieved in 71 % of patients in this age group.

In patients ≥75 years, several baseline- and treatment-related characteristics were associated with an increased risk of poor functional outcome. A recent large case series of patients with BAO, in which only a minority of patients was ≥75 years, identified similar risk factors for poor functional outcome and death [7].

The strength of this study is that BASICS was a prospective registry of consecutive patients, and therefore our results are representative for daily practice. A limitation of this study is that this was a post hoc analysis of non-randomized data, and therefore the data regarding treatment-dependent outcomes are prone to bias. Due to the prospective collection of detailed data, we were able to perform Poisson regression analyses to adjust for important confounding baseline characteristics.

We conclude that a substantial group of elderly patients survives with a good functional outcome. This study cannot answer the question which treatment option is superior in elderly patients, nor can it define an upper age limit above which treatment is no longer effective. These and other questions may be answered in the recently started BASICS trial in which patients with BAO of up to 85 years of age are randomized between intravenous thrombolysis (IVT) alone vs. IVT followed by additional intra-arterial therapy (http://www.trialregister.nl/trialreg/admin/rctview.asp?TC=2617; accessed February 1, 2012).

## Electronic supplementary material

Below is the link to the electronic supplementary material.

Supplementary material 1 (DOC 23 kb)
