# Supplementary material for: Outcomes of basilar artery occlusion in patients aged 75 years or older in the Basilar Artery International Cooperation Study
Source: J Neurol. 2012 Apr 18;259(11):2341–6. doi: 10.1007/s00415-012-6498-2 (PMC3484310; doi:10.1007/s00415-012-6498-2)
Supplement: Supplementary file 1 — Supplementary material 1 (DOC 23 kb) [file 415_2012_6498_MOESM1_ESM.doc]

**Participating centers (with number of patients and names of investigators)**

Participating centers (with number of patients and names of investigators) were as follows. Australia (6): University of Melbourne (A.M. Weber, G.A. Donnan); Belgium (21): University Hospital, Leuven (11; V. Thijs), University Hospital St. Luc, Brussels (10; A. Peeters); Brazil (18): University of Rio de Janeiro (11; G. de Freitas), University of Sao Paolo, Hospital das Clinicas (5; A.B. Conforto), Federal University of Sao Paolo (2; M. Miranda-Alves, A. Massaro); Finland (14): University of Helsinki (14; P. Ijäs, T. Bogoslovsky, P.J. Lindsberg); Germany (224): German Stroke Database (77; C. Weimar, J. Benemann, K. Kraywinkel), University Hospital Freiburg (20; C. Haverkamp), Leipzig University (15; D. Michalski), University Hospital Essen (10; C. Weimar), Medical University Hannover (8; K. Weissenborn), 6; University Hospital, Magdeburg (M. Goertler), 4; University Hospital Rostock (A. Kloth), Kliniken Neuruppin (3; A. Bitsch), Bürger Hospital, Stuttgart (3; T. Mieck), Heinrich Braun Krankenhaus, Zwickau (2; J. Machetanz), Sofien and Hufeland Hospital, Weimar (2; P. Möller), University Hospital, Ulm (2; R. Huber), Hospital Heidenheim (2; S. Kaendler), St. Elisabeth Hospital, Ravensburg (47; C. Rueckert), TEMPiS Network Bavaria (38; H. Audebert, R. Müller, B. Vatankhah), University of Munich (26; T. Pfefferkorn, T.E. Mayer), Universitätsklinikum Mannheim (19; K. Szabo), Dresden University (13; C. Disque), Klinikum Minden (2; O. Busse), University of Heidelberg (2; C. Berger, W. Hacke); Israel (19): Sheba Medical Center (19; Y. Schwammenthal, D. Orion, D. Tanne); Italy (6): University of Turin (5; M. Bergui), University of Bologna (1; E. Pozzati); Netherlands (82): St. Antonius Hospital, Nieuwegein (40; W.J. Schonewille), University Medical Center Utrecht (22; W.J. Schonewille, A. Algra, L.J. Kappelle), University Medical Center Groningen (6; G.J. Luijckx, P. Vroomen), Academic Medical Center, Amsterdam (5; M.D. Vergouwen, Y. Roos, J. Stam), Gelre Hospital (4; P. Bienfait), University Medical Center Nijmegen (3; F.E. de Leeuw), St. Elisabeth Hospital, Tilburg (1; P. de Kort), Erasmus Medical Center, Rotterdam (1; D. Dippel); Scotland (23): Southern General Hospital, Glasgow (23; T. Baird, K. Muir); Spain (25): Hospital Val d‘ Hebron, Barcelona (13; J. Pagola, M. Ribo, C. Molina), Hospital Virgen del Rocio, Sevilla (12; A. Gonzales, A. Gil-Peralta); Sweden (3): Lund University (3; B. Norrving); Switzerland (127): Inselspital, Bern (52; M. Arnold, U. Fischer, J. Gralla, H. Mattle, G. Schroth), Centre Hospitalier Universitaire Vaudois, Lausanne (39; P. Michel), University Hospital, Basel (24; S.T. Engelter, S. Wetzel, P. Lyrer), University Hospital Zurich (8; J. Gandjour, N. Michael, R. Baumgartner), Kantonsspital, St. Gallen (2; B. Tettenborn), Kantonsspital, Aarau (2; H. Hungerbuehler); United States (51): Stanford Stroke Center, Palo Alto, Calif (29; C.A. Wijman, A. Finley Caulfield, M. Lansberg, N. Schwartz, C. Venkatasubramanian), University of Texas, Houston (22; Z. Garami, S. Bogaard, F. Yatzu, J. Grotta).
